# Splicing factor Prp18p promotes genome-wide fidelity of consensus 3′-splice sites

**DOI:** 10.1093/nar/gkad968

**Published:** 2023-11-13

**Authors:** Kevin R Roy, Jason Gabunilas, Dean Neutel, Michelle Ai, Zoe Yeh, Joyce Samson, Guochang Lyu, Guillaume F Chanfreau

**Affiliations:** Department of Chemistry and Biochemistry, University of California Los Angeles, Los Angeles, CA, USA; Molecular Biology Institute, University of California Los Angeles, Los Angeles, CA, USA; Department of Chemistry and Biochemistry, University of California Los Angeles, Los Angeles, CA, USA; Department of Chemistry and Biochemistry, University of California Los Angeles, Los Angeles, CA, USA; Department of Chemistry and Biochemistry, University of California Los Angeles, Los Angeles, CA, USA; Department of Chemistry and Biochemistry, University of California Los Angeles, Los Angeles, CA, USA; Department of Chemistry and Biochemistry, University of California Los Angeles, Los Angeles, CA, USA; Department of Chemistry and Biochemistry, University of California Los Angeles, Los Angeles, CA, USA; Department of Chemistry and Biochemistry, University of California Los Angeles, Los Angeles, CA, USA; Molecular Biology Institute, University of California Los Angeles, Los Angeles, CA, USA

## Abstract

The fidelity of splice site selection is critical for proper gene expression. In particular, proper recognition of 3′-splice site (3′SS) sequences by the spliceosome is challenging considering the low complexity of the 3′SS consensus sequence YAG. Here, we show that absence of the Prp18p splicing factor results in genome-wide activation of alternative 3′SS in *S. cerevisiae*, including highly unusual non-YAG sequences. Usage of these non-canonical 3′SS in the absence of Prp18p is enhanced by upstream poly(U) tracts and by their potential to interact with the first intronic nucleoside, allowing them to dock in the spliceosome active site instead of the normal 3′SS. The role of Prp18p in 3′SS fidelity is facilitated by interactions with Slu7p and Prp8p, but cannot be fulfilled by Slu7p, identifying a unique role for Prp18p in 3′SS fidelity. This fidelity function is synergized by the downstream proofreading activity of the Prp22p helicase, but is independent from another late splicing helicase, Prp43p. Our results show that spliceosomes exhibit remarkably relaxed 3′SS sequence usage in the absence of Prp18p and identify a network of spliceosomal interactions centered on Prp18p which are required to promote the fidelity of the recognition of consensus 3′SS sequences.

## Graphical Abstract

**Graphical Abstract:**
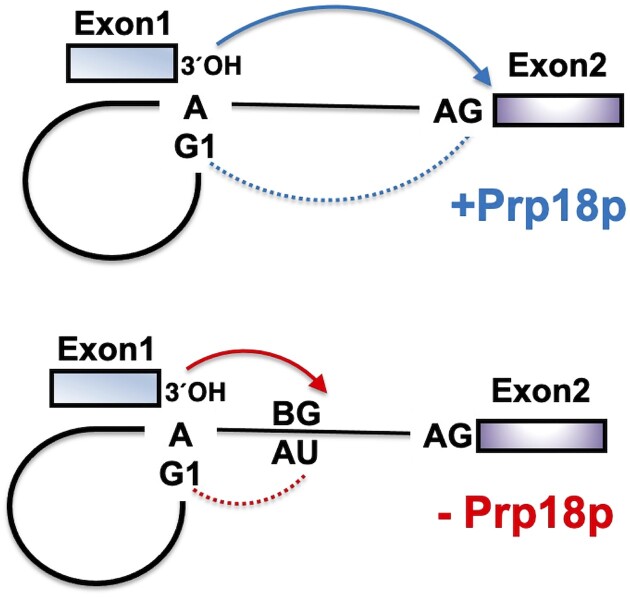

## Introduction

Accurate splice site selection is critical for proper gene expression, as errors in splicing are known to cause a large number of genetic diseases and cancer (1). However, proper splice site selection offers a major mechanistic challenge to the spliceosome in part because of the simplicity of splicing signals, as multiple near-consensus ‘incorrect’ sites often exist at the vicinity of the ‘correct’ site. This is especially problematic for 3′-splice sites (3′SS) given their very simple consensus sequence YAG (Y = C or U). Although the 3′SS is typically selected prior to the first chemical step during metazoan splicing (2), the second catalytic step of pre-mRNA splicing offers additional opportunity to further recognize the appropriate 3′SS. Genetic and structural studies have shown that base pairing interactions between the 5′SS and 3′SS nucleosides play a role in the efficiency of the second step of splicing and in 3′SS recognition (3–6). Following an ATP-dependent rearrangement of the spliceosome after the first catalytic step (7,8), Slu7p, Prp18p and Prp22p sequentially join the spliceosome to facilitate the second catalytic step (9,10). In particular, Prp18p and Slu7p are necessary for the efficient docking of the 3′SS into the spliceosomal active site (5). In addition to Prp18p and Slu7p, the alpha finger of Prp8p is thought to facilitate selection of *bona fide* 3′SS sequences, as Gln1948 of Prp8p interacts specifically with the pyrimidine base at position –3 (5). The newly identified splicing factor Fyv6p has also been shown to facilitate the second step of splicing and to promote usage of *bona fide* 3′SS (11). Finally, Prp22p remodels pre-mRNA to promote sampling of alternative 3′SS (12), and proofreads the ligated exons in an ATP-dependent manner following the second transesterification reaction (13).

Although Prp18p is positioned at the core of the spliceosome during the second chemical step, its function in the fidelity of recognition of 3′SS sequences is not well understood. A previous cryo-EM study which identified particles lacking Prp18p showed that the 3′SS is not stably docked in the active site (5). However these particles also lacked Slu7p, making it difficult to assess the specific contribution of Prp18p. Deletion of the gene encoding Prp18p results in a severe growth defect (14), suggesting that Prp18p plays a major role on the efficiency of splicing *in vivo*. However, Prp18p and Slu7p are not absolutely required for the second splicing step of all pre-mRNAs, as they are dispensable for splicing of substrates in which the distance from the branchpoint to the 3′SS is short (15,16). Previous work using splicing reporters has shown that Prp18p plays a role in stabilizing the interactions between exonic nucleotides and nucleotides of the U5 snRNA loop1 (17). The role of Prp18p in stabilizing splicing intermediates for the second step of splicing (17,18) and its position near the 3′SS during the second step (5,8) with the conserved region loop of Prp18p positioned near nucleosides –3 and –4 of the 3′SS sequence suggests that it may also contribute to the fidelity of selection of YAG 3′SS. In agreement with this hypothesis, a previous study showed that the absence of Prp18p can favor selection of certain upstream 3′SS *in vivo* (19). However, the global role of Prp18p in promoting splicing efficiency and fidelity has not been investigated. To assess the impact of Prp18p on splicing and 3′SS selection genome-wide, we performed transcriptome analysis of cells lacking Prp18p and found that the absence of Prp18p resulted in a global reduction of splicing efficiency of non-ribosomal protein genes and in a loss of splicing fidelity with widespread activation of 3′SS whose sequence deviate from the consensus. We show that the role of Prp18p in promoting 3′SS sequence fidelity depends on its recruitment by Slu7p and on interactions with the RNase H domain of Prp8p but that the role of Prp18p in promoting proper 3′SS sequences fidelity cannot be fulfilled by an excess of Slu7p. We also show that this 3′-SS fidelity is synergized by the proofreading activity of the Prp22p helicase which acts downstream of Prp18p. These results identify the primary mechanisms by which *Saccharomyces cerevisiae* spliceosomal factors promote recognition of 3′SS sequences and point to a unique role for Prp18p in facilitating the docking of 3′SS sequences harboring the proper YAG sequence in the active site of the spliceosome *in vivo*.

## Materials and methods

### Yeast strain, plasmid construction and protein assays

Yeast strain construction, RT-PCR analysis, molecular cloning, restriction enzyme-based plasmid construction, site-directed mutagenesis and spot dilution assays were performed as previously described (20–22). Construction of the PRP18/pUG35 and PRP18/YEp24 base plasmids was accomplished using Gibson assembly (23) (New England Biolabs #E2611). Unless otherwise indicated in the figure legends, all strains were derived from the BY4741 background. The *PRP8* genomic mutations were introduced using the CRISPR-Cas system as described (24). The *SLU7* and *PRP22*/*PRP43* mutant strains and plasmids were generously provided by Beate Schwer (Cornell Weill Medical College) and Jonathan Staley (U. Chicago), respectively. For the western blot analysis used to verify Prp18p constructs expression, proteins were extracted using the TCA protocol described in (25). Western blot analysis was performed using affinity purified anti-Prp18 antibodies (15) generously provided by B. Schwer (Cornell Weill Medical College).

### Yeast growth, RNA preparation and sequencing

Yeast strains were grown and harvested as previously described (20,21). For heat-sensitive mutant strains, cell cultures were first grown to exponential phase at 25°C, a portion harvested, and the remaining culture shifted to 37°C and incubated at that temperature for 1 h. For cold-sensitive mutant strains, cell cultures were first grown to exponential phase at 30°C, a portion harvested, and the remaining culture split and shifted to 16°C or 25°C and incubated at those temperatures for 2 h. For the RNA-Seq analysis, *upf1Δ::HIS3MX6* and *upf1Δ::HIS3MX prp18Δ::kanMX6* colonies were inoculated for overnight growth in YPD, seeded in 50 ml fresh YPD the following day at OD_600 nm_ 0.05 and grown to mid-log phase OD_600 nm_ of 0.6. Samples were harvested by spinning down at 3k rpm for 5 min, flash-frozen in liquid nitrogen, and total RNA was purified by standard phenol–chloroform extraction as described previously (21). Fragmented RNA seq libraries were prepared using the Illumina TruSeq stranded mRNA library prep kit. 2 × 100 bp sequencing on the HiSeq 2000 and subsequent demultiplexing were performed by Macrogen/Axeq (South Korea).

### Computational methods

A detailed description of all computational steps is outlined in the Supplementary Methods. All scripts for the Comparison of Multiple alignment Programs for Alternative Splice Site discovery (COMPASS) read processing pipeline are available on Zenodo (https://doi.org/10.5281/zenodo.8387420). The most recent COMPASS release can be found on GitHub (https://github.com/k-roy/COMPASS). COMPASS includes the following steps: pre-processing of raw reads, alignment with multiple aligners, post-alignment processing, selection of the best scoring alignment for each read, and quality filtering of junctions. The aligners STAR (26) and BBMap [https://sourceforge.net/projects/bbmap, https://www.osti.gov/biblio/1241166] were used in this study. Data and scripts used for downstream analyses can be found in the zenodo.8387420 release subfolder ‘./yeast_Prp18’.

### Splicing efficiency (SE) and fraction of annotated splicing (FAnS) calculation

We calculated splicing efficiency (SE) as the ratio of the reads mapping to each splice junction over the sum of all reads mapping to both spliced and unspliced junctions for each intron. Unspliced mRNA harbors both 5′SS and 3′SS junctions so the combined read counts for these junctions are divided by 2. For each alternative splice junction, we calculated a fraction of annotated splicing (FAnS) as the ratio of the reads mapping to the junction relative to the reads mapping to the annotated junction. For both SE and FAnS, we also calculated a *prp18*Δ effect by taking the ratio of the *prp18*Δ *upf1*Δ strain relative to the *upf1*Δ strain.

### Intron sequence and structure prediction analyses

For each 3′ SS, the intronic sequence was examined for the sequence most closely matching the branchpoint consensus TACTAAC, with each mismatch penalized by a score of 1, and an additional penalty of 2 was given if the conserved branched adenosine (underlined) was not present. The sequence with the lowest mismatch score was assigned as the most likely branchpoint, and ties were broken by taking the branchpoint with the shortest distance to the 3′SS. The linear branchpoint (BP)–3′SS distance was defined as the number of nucleotides between the branching A of the BP and the 3′SS, such that TACTA**A**CACNNNN|TAG would be a distance of 10 nt, in line with previous conventions (27,28). See COMPASS_analyze_splice_junction_profiles_for_individual_ samples.py.

Effective branchpoint (BP)–3′SS distances and RNA tri-nucleotide accessibilities in the BP–3′SS region were calculated similarly to a previous report (28). For each annotated and alternative 3′SS, four windows of increasing size with lengths *n* + 5, *n*+ 10, *n* + 15 and *n* + 20 respectively, where *n* is the distance between the BP and 3′SS, were folded with rnafold version 2.4.6 with options –partfunc –constraint (29). The –constraint option enables keeping the branch sequence and 8 nt downstream unpaired as this sequence has been proposed to be kept in an unpaired configuration by the spliceosome(28). The effective distance from the branchpoint is determined from the lowest energy structure from the parens/bracket notation from rnafold. The effective distance of any base X is calculated by examining the fold in between base X and the branchpoint. The number of bases between base X and the branchpoint which are paired with other bases between base X are subtracted from the linear distance, with bases involved in loops treated as paired. The –partfunc option of rnafold calculates the partition function and base pairing probability matrix which are written to dp.ps files. For each base, the probability of being paired with each other base is summed to give a probability of each base being in a base pair. The accessibility of each base was then calculated as 1 minus this probability. The accessibility value for each trinucleotide in the region from 7 nt downstream the branchpoint to 5 nt downstream the splice site was calculated as the average of the accessibility values of the three bases averaged over the four windows. For the comprehensive analysis of each annotated intron where sites are analyzed as used versus unused as shown in Figure 3C, 50 nt were added to the end of the annotated 3′SS. See COMPASS_write_BP_3SS_to_fasta.py and COMPASS_analyze_BP_3SS_RNA_folds_for_annotated_introns.py. The polyU score of each trinucleotide in the region from 7 nt downstream the branchpoint to 5 nt downstream the annotated splice site was calculated using the position weight matrix scores (PWMS) of the yeast polyU tract according to (30).

### RT-PCR analysis and quantification of alternative splice site usage

RT-PCR analysis of alternative splice site usage using fluorescently labeled primers was performed as described (31). Quantification of the bands corresponding to each isoform was measured using ImageJ 1.53K. To determine the intensity of each band, three separate boxes were created and the average intensity of the three was used as the band intensity. The background intensity for each lane was subtracted from the average band intensity in order to remove background bias. Samples of interest were compared in triplicate using two-tailed t-tests.

## Results

### Absence of Prp18p reduces splicing efficiency of introns for primarily non-ribosomal protein genes

**Figure 1.**
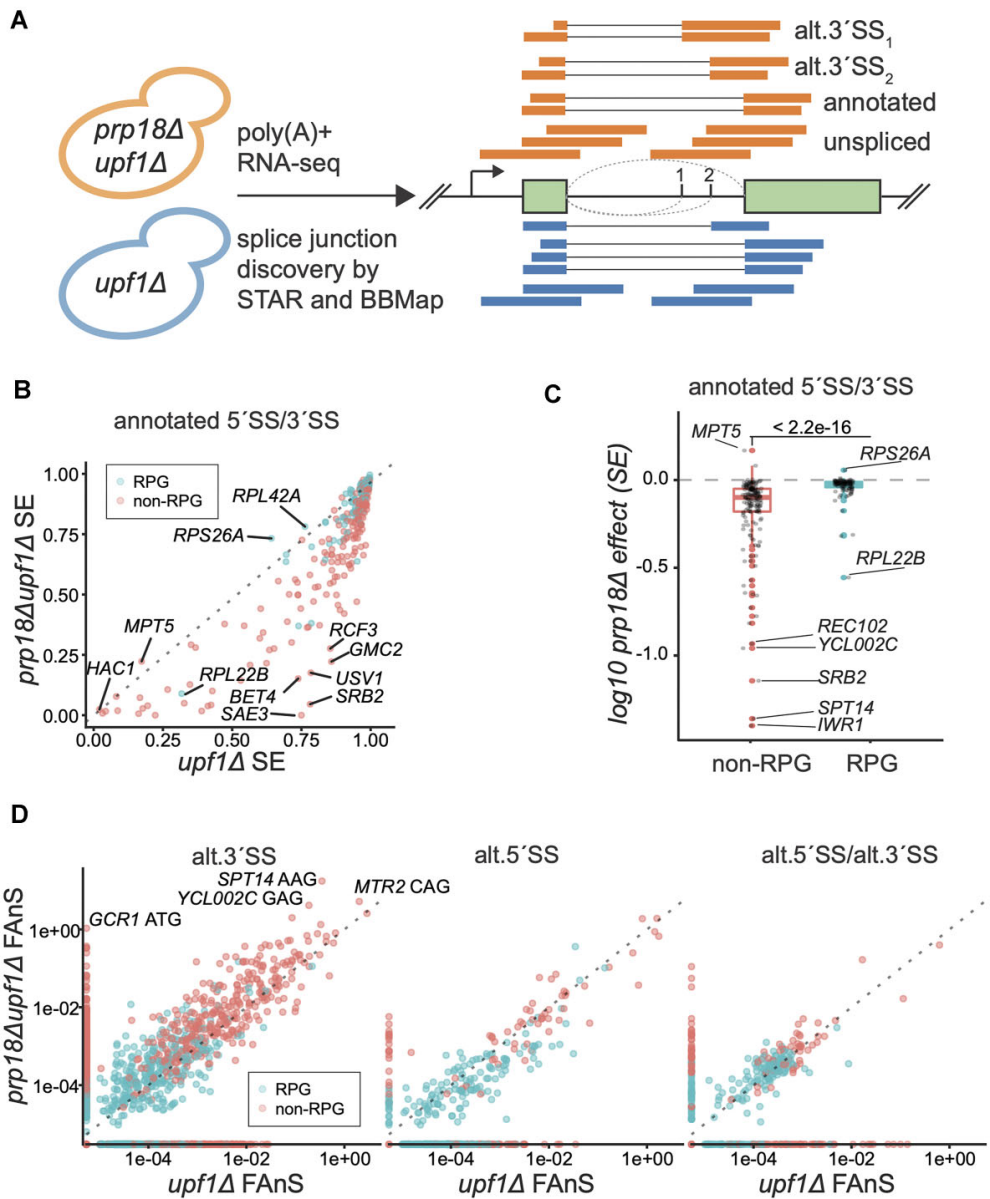
Global impact of Prp18p on annotated and alternative splicing events. (**A**) Diagram describing the workflow for identifying alternative splice junctions accumulating in *prp18Δupf1Δ* versus *upf1Δ*. Alignments generated by an annotation-guided aligner (STAR) and an annotation/motif-agnostic aligner (BBMap) are processed by COMPASS to identify the optimal alignment for each read with a gapped alignment. (**B**) Splicing efficiency (SE) in *prp18Δupf1Δ* versus *upf1Δ* for annotated introns with ribosomal protein genes (RPGs) in blue and non-RPGs in red. (**C**) Effect of Prp18 inactivation of the splicing efficiency (SE) of intron containing genes separated in non-RPGs (red) and RPGs (blue) classes. Shown is a boxplot of the *prp18Δ* effect on SE (*prp18Δupf1Δ* SE/ *upf1Δ* SE) for annotated splice junctions. Log10 less than zero represents decreased splicing in the absence of Prp18p. *P*-values are based on two-tailed *t*-test analysis. (**D**) Fraction of annotated splicing (FAnS) for ann.5′SS/alt.3′SS (left), alt.5′SS/ ann.3′SS (middle), or alt.5′SS/alt.3′SS (right). Points on the axes indicate junctions for which splicing is not detected in the opposing strain (note the log scale).

Prp18p was previously shown to suppress the usage of an unusual AUG 3′SS in the *GCR1* pre-mRNA (19). However, the genome-wide role of Prp18p in splicing efficiency and promoting 3′SS fidelity is currently unknown. To globally analyze the impact of Prp18p on splicing, we performed RNA sequencing on cells expressing wild-type spliceosomes or lacking Prp18p (*prp18Δ*) in a genetic background deficient for nonsense-mediated mRNA decay (NMD) through inactivation of the NMD helicase Upf1p (Figure 1A). This genetic background was chosen to stabilize alternatively spliced mRNAs harboring premature termination codons (PTCs) (19), allowing a more direct analysis of the spliced transcript production without confounding effects of differential isoforms stability. We obtained 60–100 million uniquely mapped 100 bp reads for each of the three replicates for each strain. 2–3% of all reads mapped with gapped alignments, and 93–95% of the gapped alignments corresponded to annotated splicing junctions.

We first analyzed the splicing efficiency (SE) for each annotated splice junction in the presence and absence of Prp18p (see Materials and methods). Consistent with a previous analysis using tiling microarrays (14), inactivation of Prp18p significantly reduced SE genome-wide (Figure 1B). As described in previous studies (32,33), ribosomal protein genes (RPGs) pre-mRNAs exhibited higher SE relative to non-RPGs in the control strain for the annotated splice sites (Figure 1B). To further assess the impact of Prp18p on splicing, we calculated a *prp18Δ*-effect index for SE, where a positive *prp18Δ* effect indicates increased splicing in *prp18Δ*, and negative indicates decreased splicing (see Methods). As expected, the loss of Prp18 had an overall negative impact on SE for all annotated junctions (Figure 1B, C, Supplementary Figure S1). However, there was a much greater negative impact on non-RPGs pre-mRNAs SE relative to RPGs (Figure 1C), showing that the splicing of RPG pre-mRNAs is more resilient to the loss of Prp18p than that of non-RPGs.

### A multi-aligner bioinformatics approach reveals widespread usage of alternative splice sites in the absence of Prp18p

Next, we analyzed alternative splice junctions that arise in the absence of Prp18p. We noticed that STAR (26), a commonly used RNA-seq aligner, failed to map reads correctly for an alternative 3′SS that used a non-canonical 3′SS sequences (e.g. AUG for the *MUD1* intron). By contrast, we found that BBMap, a general-purpose aligner agnostic to splice motifs [https://sourceforge.net/projects/bbmap/, https://www.osti.gov/biblio/1241166], was able to map these reads correctly but often failed to map reads with short overhangs at annotated junctions where STAR performed well. To combine the strengths of each aligner, we systematically compared STAR and BBMap alignments for each read harboring a potential splice-junction and selected the alignment exhibiting the fewest mismatches to the reference. We further removed likely false positive junctions through quality filtering of the STAR-BBMap integrated set of junctions to obtain a high-confidence set of alternative and novel junctions (Supplementary Tables S1–S5).

To analyze how splicing patterns at individual genes shift in the absence of Prp18p, we calculated the Fraction of Annotated Splicing (FAnS) for all alternative splicing junctions in the presence and absence of Prp18p. The FAnS ratio reports the relative abundance of an alternative splicing event compared to the canonical, annotated main spliced isoform. RPGs pre-mRNAs constitute 90% of *S. cerevisiae* intron-containing mRNAs by transcript abundance (32) even though they represent only 1/3 of all intron-containing genes (ICGs). Overall, RPGs exhibited *c.a*. 6-fold more annotated junctions reads than non-RPGs, but only 1.63-fold more alternative junctions reads in RPGs versus non-RPGs in the *upf1Δ* strain, and equal reads (1.01:1) in the *upf1Δprp18Δ* strain (Supplementary Figure S1). These observations show that the alternative splicing events detected do not simply arise due to spliceosomal noise that scales with expression levels, but are governed by specific features in ICGs that control splicing fidelity, as suggested previously (34). Consistent with this, alternative junctions in non-RPGs showed significantly higher FAnS than those in RPGs (Figure 1D). Importantly, the FAnS calculated from our sequencing data set showed a high level of concordance with the FAnS calculated from a study examining alternative AG sites usage in a *upf1Δ* strain (34) (Supplementary Figure S2).

The absence of Prp18p globally activated alternative 3′SS usage and inhibited alternative 5′SS usage relative to annotated sites (Figure 1D). In particular, the absence of Prp18p revealed a large number of alternative 3′SS in non-RPG transcripts, which were virtually undetectable in the *upf1Δ* strain (Figure 1D, left panel), including the *GCR1* AUG 3′ SS site previously described (19). While for many splicing events, the increase in FAnS is largely driven by a decrease in annotated SE, many splice sites exhibited an increased SE in the absence of Prp18p (8 annotated sites, 294 alt.3′SS, 15 alt.5′SS, 47 alt.5′SS/alt.3′SS; Supplementary Figure S3). Remarkably, 9 splice isoforms that used alternative 3′SS in 6 genes exhibited greater spliced mRNA abundance than the mRNA produced from the annotated sites (*GCR1*, *MTR2*, *REC102*, *REC107*, *SPT14*, *YCL002C*; Supplementary Table S5), and 15% of all introns (42/280) exhibited at least 10% alt.3′SS usage (Supplementary Table S5). These data show that Prp18p promotes global recognition of *bona fide* 3′SS over competing alternative sites.

### Spliceosomes exhibit widespread relaxed fidelity towards non-YAG 3′SS sequences in the absence of Prp18p

**Figure 2.**
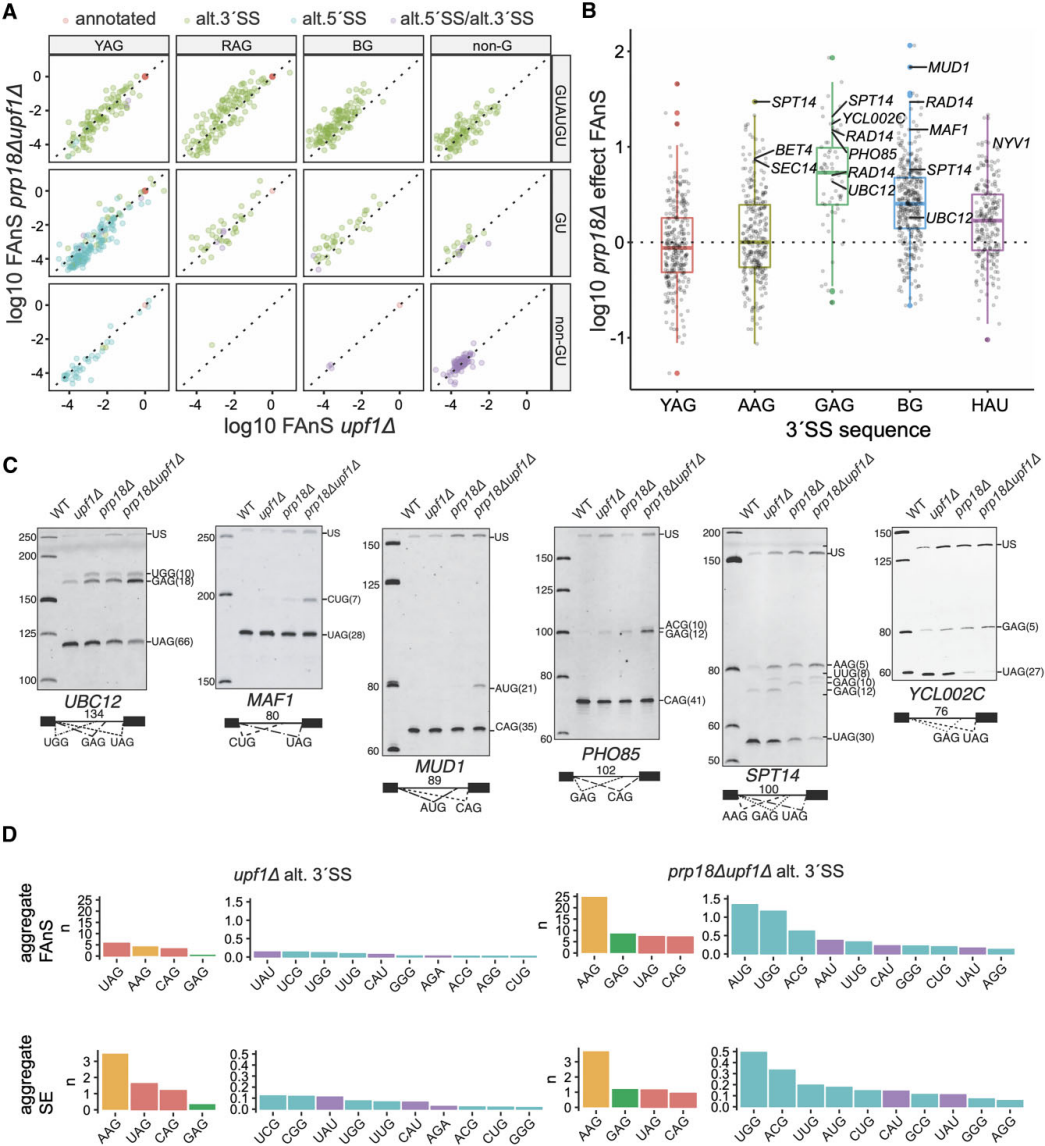
Widespread activation of non-canonical 3′SS in the absence of Prp18p. (**A**) Fraction of annotated splicing (FAnS) and (**B**) boxplots of the *prp18Δ* effect (*prp18Δupf1Δ* FAnS/ *upf1Δ* FAnS) for different classes of 3′SS motifs. (**C)** Selected junctions validated by RT-PCR with Cy3-labeled primers and polyacrylamide gel electrophoresis. The numbers in each intron diagram represent the length of the annotated intron. The numbers beside each 3′SS sequence on the gel represent distance (in bp) from the branchpoint (BP) to the indicated 3′SS. Note that each of these alternative junctions as well as those in Supplementary Figure S8 is labeled in the *prp18Δ* effect boxplots in panel B. (**D**) Aggregate usage of each motif class calculated as the sum of FAnS or SE for all alternative sites in each class. Salmon = YAG; green = GAG; orange = AAG; cyan = BG; purple = HAU or AGA. Abbreviations: R = A or G; Y = C or U; H = A, C or U.

To identify the genomic determinants for alternative 3′SS activation in the absence of Prp18p, we analyzed FAnS as a function of the sequence of these alternative 3′SS sequence. These 3′SS motifs could be broadly divided into YAG, RAG, BG (where R = A or G; B = C, G or U), and non-G motifs, with roughly equal numbers of junctions (126 YAG, 166 RAG, 156 BG, 115 non-G, 6-reads minimum, Supplementary Table S2, Figure 2A, B). The majority of these sites had not been detected in previous transcriptome-wide analyses of alternative splicing, likely a consequence of the low usage of these sites when Prp18p is functional and because of biases towards AG/AC introns in the mapping algorithms previously used(19,34–36). Strikingly, *prp18Δ* spliceosomes tended to specifically activate alternative 3′SS harboring non-YAG/non-AAG motifs, with the BG and HAU (H = A, C or U) sequences being the most frequent non-canonical sequences activated in the absence of Prp18p (Figure 2A, B). GAG and BG 3′SS were the most upregulated in the absence of Prp18p, with most alternative YAG sites instead being down-regulated (Figure 2A, B). We confirmed the activation of the non-canonical 3′SS by performing targeted RT-PCR on selected transcripts, including non-consensus 3′SS UGG in *UBC12*, CUG in *MAF1*, AUG in *MUD1*, ACG in *PHO85* and UUG in *SPT14* (Figure 2C). We also confirmed usage of a highly unusual CAU 3′SS for *NYV1* (see below). For *SPT14* and *YCL002C*, RT-PCR confirmed the near-complete loss of the annotated spliced isoforms with concomitant activation of alternative upstream RAG sites (Figure 2C).

We next analyzed the aggregate usage for each 3′SS sequence genome-wide by summing SE and FAnS across transcripts, and found AAG to be the most utilized alternative 3′SS sequence by total SE (Figure 2D). Although the overall SE for AAG was roughly the same in both strains, the FAnS at AAG sites was substantially higher in the absence of Prp18p, suggesting a preferential loss of annotated 3′SS usage in the absence of Prp18p (Figure 2D, Supplementary Figure S4). While AAG spliced junctions outnumber GAG junctions, the SE for GAG increased by a larger extent in the absence of Prp18p relative to AAG (Figure 2D). AUG, ACG, UGG and UUG were the most efficiently used non canonical BG sites, while CAU and UAU were the most efficiently used non-G sites (Figure 2D). In summary, these results demonstrate that in the absence of Prp18p, the spliceosome can utilize 3′SS lacking the consensus A or G nucleosides that are the hallmark of most 3′SS. In other words, spliceosomes exhibit remarkably relaxed fidelity in the absence of Prp18p, with a reduced ability to discriminate YAG from non-YAG sequences.

### Prp18p prevents activation of branchpoint-proximal 3′SS and of non-YAG 3′SS sequences

**Figure 3.**
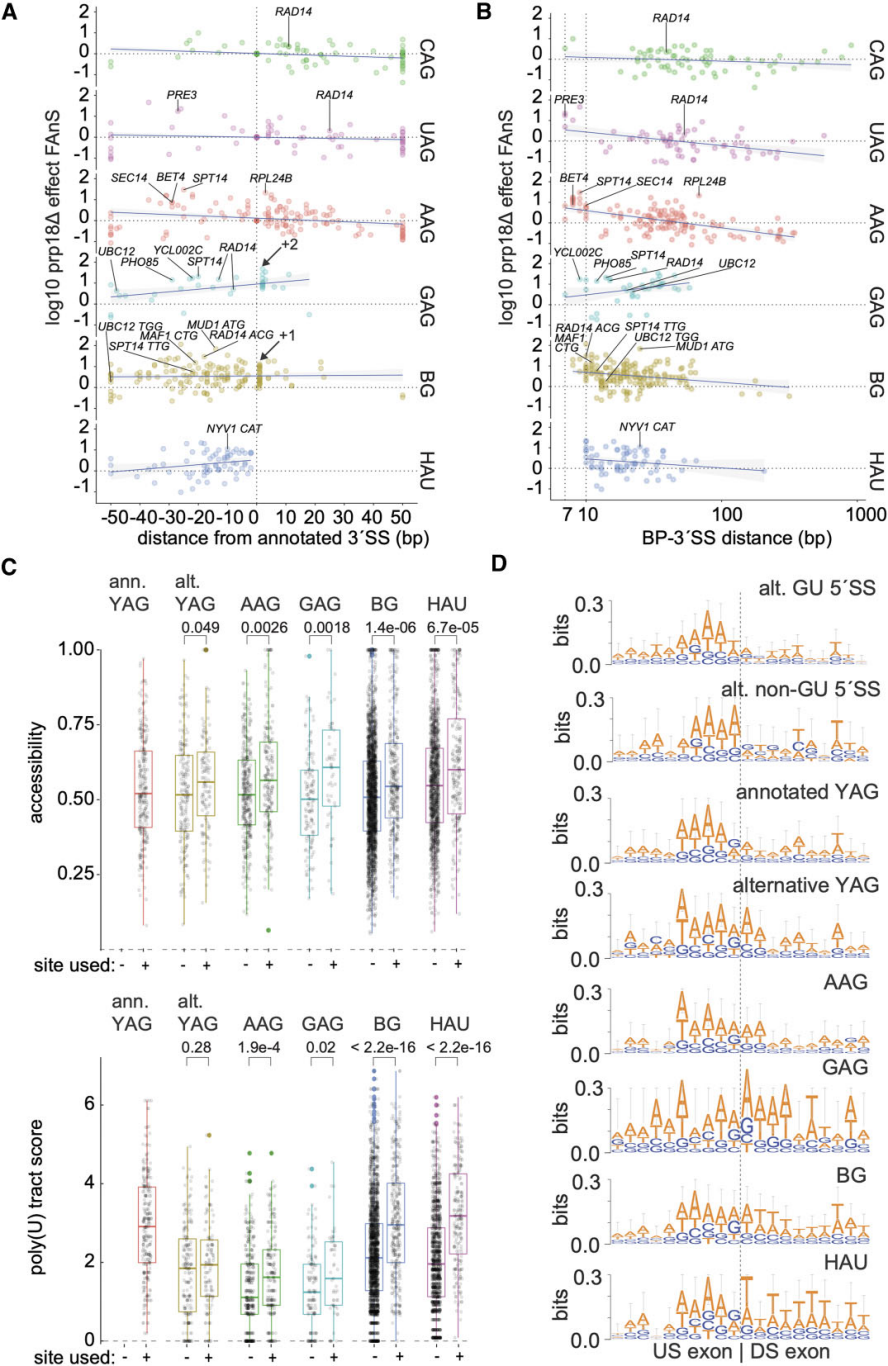
Adjacent sequences and structural contexts modulate alternative 3′SS usage. (**A**) *prp18Δ* effect (*prp18Δupf1Δ* SE/ *upf1Δ* SE) plotted by the alternative to annotated 3′SS distances for each motif class, where negative numbers represent upstream alt.3′SS. The horizontal line denotes neutral effect of Prp18p. The indicated +1 and +2 positions from the annotated site denote positions enriched for BG and GAG non-canonical splicing motifs, respectively. (**B**) *prp18Δ* effect as defined in (A) but plotted by the distance from the branchpoint (BP) to the 3′SS. Note the log scale for the x-axis. (**C**) Boxplots of 3′SS accessibility and poly(U) tract scores separated by whether the 3′SS gives detectable splicing (+) or not (–) in the *prp18Δupf1Δ* strain. The accessibility of each trinucleotide sequence from 8 nt downstream the branchpoint to 50 nt downstream of the annotated site was calculated as the averaged probability of each base in the 3′SS being unpaired (see Materials and methods). *P*-values are based on two-tailed *t*-test analysis. (**D**) Motif enrichment for the 10 nucleotides upstream and downstream of each junction for each indicated 5′SS and 3′SS class. The vertical dotted line denotes the exon-exon junction.

We next analyzed the features of 3′SS sequences activated in the absence of Prp18p, first focusing on their location relative to the annotated 3′SS and branchpoint sequences (Figure 3A, B). Plotting the position of alternative 3′SS by distance to the annotated 3′SS revealed that alternative YAG and AAG 3′SS tend to be downstream, while GAG and non-AG sites tend to be upstream of the annotated 3′SS (Figure 3A). The main exceptions to this trend were a group of GAG and BG alternative 3′SS located 2 and 1 nucleotides downstream of the annotated one (Figure 3A). This observation is consistent with the model that spliceosomes lacking Prp18p may exhibit ‘slippage’ when suboptimal 3′SS sequences are located immediately adjacent to the normal 3′SS. Many alternative 3′SS used in the absence of Prp18p were located less than 10 nt from the annotated branchpoint (Figure 3B). Whereas the shortest known naturally occurring BP-3′SS distance in budding yeast is 10 nt, we detected 25 cases of alternative 3′SS positioned within 10 nt of the branchpoint, with the shortest distance of 7 nt occurring 7 times (*DYN2*, *PRE3*, *NCE101*, *RUB1*, *RPL40B*, *RPS18B*, *RPS27A*; Figure 3B). These sites were utilized at substantially increased levels in the absence of Prp18p, consistent with spliceosome structural studies that predicted that Prp18p acts a molecular ruler imposing a minimum BP-3′SS distance (5).

While we observed a weak relationship between alternative 3′SS efficiency and BP-3′SS distance in the *upf1*Δ control strain, a stronger negative relationship between alternative 3′SS efficiency and BP-3′SS distance emerged in the absence of Prp18p (Supplementary Figure S5). This effect was strongest for AG sites, likely owing to the increased numbers of sites utilized downstream of the annotated 3′SS (Figure 3A, B). While YAG sites downstream of the annotated sites tended to be downregulated in the absence of Prp18p, downstream RAG and BG sites were more often upregulated relative to the annotated sites (Figure 3A). This suggests that the increased utilization of non-consensus 3′SS is not entirely due to Prp18p-deficient spliceosomes favoring shorter BP-3′SS distances, but rather indicative of an independent function for Prp18p in YAG sequence selection. Supporting this model, we noticed an enrichment of upregulated NGG sites at +1 and GAG sites at +2 nt downstream of the annotated 3′SS, where the underlined G represents the G_-1_ of the annotated YAG 3′SS (Figure 3A; NBG are NGG sites at the +1 position due to the invariant G in annotated sites).

Accessibility of 3′SS within the context of RNA secondary structure can play a major role in determining the primary 3′SS used (i.e. the annotated site); (28,37). To test whether this impacts alternative 3′SS usage globally, we analyzed accessibility of alternative 3′SS motifs (i.e. the probability of the 3′SS not being included in a secondary structure) in a window spanning 7 nt downstream of the BP to 50 nt downstream of the annotated 3′SS. We then separated sites in two groups, according to whether they were detected as alternative 3′SS in either strain. Consistently, alternative 3′SS motifs that are utilized tended to be more accessible than those not utilized, and surprisingly, even more accessible than the annotated 3′SS (Figure 3C). Together these data support a model in which 3′SS accessibility influences its utilization by the spliceosome, consistent with previous studies (28).

### Poly(U) tract strength and exonic adenosines promote usage of alternative and non-YAG 3′SS

Next we examined the relationship between poly(U) tracts and 3′SS usage, and calculated a poly(U) score based on position weight matrix scores (PWMS) derived from all annotated 3′SS, as defined previously (30). We found that RAG, BG and HAU alternative 3′SS sites that are utilized exhibited substantially greater poly(U) tract scores than non-utilized sites (Figure 3C). This impact of poly(U) sequence was specific to non-consensus 3′SS sequences, as it was not detected for alternative YAG sites (Figure 3C). This observation suggests that activation of unconventional 3′SS sequences requires the presence of strong poly(U) tract upstream. As alternative 3′SS are in competition with annotated ones, we examined how poly(U) tract scores at the annotated sites impact the splicing efficiency (SE) at alternative 3′SS. Strikingly, we observed a significant negative correlation between the poly(U) tract scores of annotated 3′SS and SE of alternative 3′SS (Supplementary Figure S6a). This was true even when analyzing RPG and non-RPGs separately to account for the observation that RPGs tend to have much stronger poly(U) tracts (Supplementary Figure S6a). Interestingly, BG and HAU alternative 3′SS exhibited greater poly(U) tract scores relative to the annotated sites in RPG versus non-RPGs (Supplementary Figure S6b), indicating greater dependency of non-AG sites on a strong poly(U) tract in the context of RPG splicing (Supplementary Figure S6b). These differences are significant in light of the fact that GAG sites tend to have weaker poly(U) tracts relative to BG and HAU despite also being predominantly upstream of annotated sites (Figure 3C).

Previous work has shown that adenosines in both exons can promote the efficiency of the second step via interactions with the uridine-rich loop 1 of U5 snRNA, and that these interactions are particularly important in the absence of fully functional Prp18p (17,18). Consistent with these targeted analyses, sequence logo analysis of the alternative 5′SS indicated an enrichment of adenosines in the first 4–5 nt upstream, similar to that observed at annotated 5′SS (Figure 3D). While annotated 3′SS did not show any enrichment for adenosine in the downstream exons, alternative AG 3′SS showed enrichment for A at the +1 and +2 positions, with GAG showing the strongest enrichment from +1 to +4 of the pseudo-exon2 sequences (Figure 3D). The enrichment for U downstream of BG and HAU is likely due in part to the presence of poly(U) tracts upstream of the annotated site, as the majority of BG and HAU sites are found within 20 nt upstream of the annotated site, thus positioned very closely upstream from the poly(U) tract regions preceding the annotated 3′SS. Instead, GAG sites exhibit stronger enrichment of downstream pseudo-exonic adenosines, showing that different non-canonical motifs may rely on different features for efficient usage by the spliceosome (Figure 3D). Overall, these results suggest that the utilization of non-consensus 3′SS sites requires a combination of intronic and pseudo-exonic features to compete effectively with the annotated sites.

### Mutations in the conserved loop and helix2 of Prp18p impair 3′SS sequence fidelity

**Figure 4.**
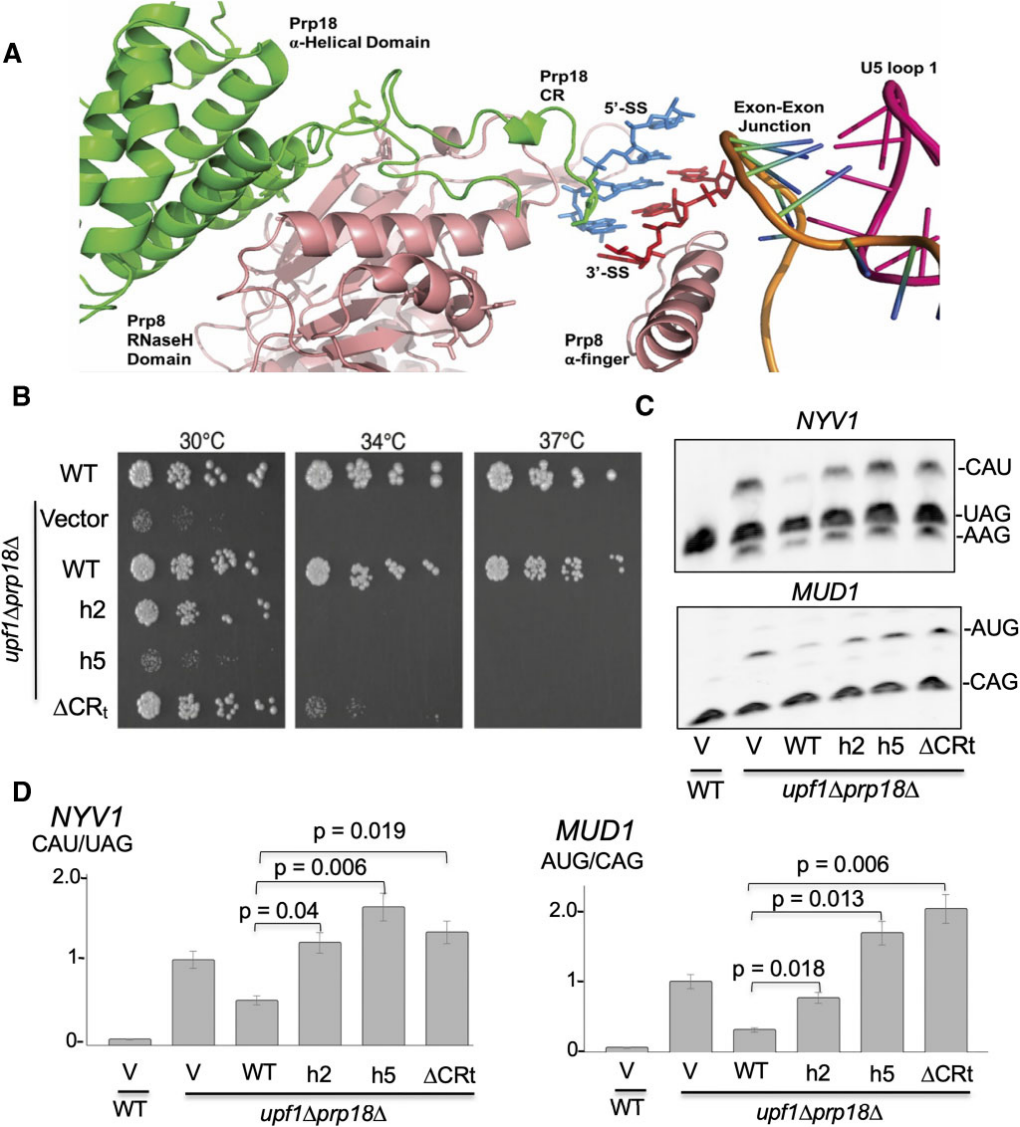
Contributions of the structural domains of Prp18p to 3′-splice site sequence recognition. (**A**) Structure of the active site of the post-catalytic spliceosome with domains of Prp18p (green), Prp8p (salmon), the 5′-SS (blue), the 3′-SS (red), the ligated exons (orange/blue/green), and the loop 1 of the U5 snRNA (pink). (**B**) Spot dilution growth assay for wild-type and *prp18*Δ*upf1*Δ strains complemented with various *PRP18* plasmids: empty pUG35 (vector) or derivatives expressing wild- type *PRP18*, a helix 2 mutant (*prp18-h2*), a helix 5 mutant (*prp18-h5*), or a mutant in which the conserved loop is completely deleted (*prp18-*ΔCRt). Strains were grown to exponential phase in CSM-URA media then serially diluted in 3-fold spot dilutions onto CSM-URA plates and incubated at the indicated temperatures for 3 days. (**C**) RT-PCR analysis of *NYV1* and *MUD1* alternative splicing in wild-type and *prp18*Δ*upf1*Δ strains complemented with an empty vector or plasmids expressing wild-type *PRP18*, a helix 2 mutant (*prp18-h2*), a helix 5 mutant (*prp18-h5*), or a mutant in which the conserved loop is completely deleted (*prp18-*ΔCRt). (**D**) Quantification of the usage of the major alternative 3′SS of *NYV1* and *MUD1* relative to the main 3′SS in arbitrary units. The y-axis indicates the ratio of intensities of the bands for the non-canonical site versus the canonical site, normalized to 1 for the *prp18*Δ*upf1*Δ strain. Error bars represent the standard deviation for three independent biological replicates. *P*-values are based on *t*-test analysis (see Materials and methods).

We next investigated the structural domains and interactions that allow Prp18p to promote the fidelity of recognition of YAG 3′SS. *S. cerevisiae* Prp18p contains five α-helices interspersed with short loop regions, including a loop connecting helices 4 and 5 that contains a highly conserved region (CR) of 25 amino acids (Figure 4A; Supplementary Figure S7) (38,39). Cryo-EM structure of the post-catalytic spliceosome revealed that the Prp18p α-helical domain interfaces with the RNase H-like domain of Prp8p at the spliceosome periphery while the conserved loop juts into the active site and is positioned near nucleosides -3 and -4 of the 3′SS via a tunnel going through Prp8p (5). This arrangement is proposed to stabilize the core spliceosome, while the Prp18p CR along with the α-finger domain of Prp8p forms a channel within the active site through which the 3′SS can be positioned for exon ligation (5) (Figure 4A). These observations suggest that the CR loop of Prp18p might promote the fidelity of the second step of splicing through its interaction with the 3′SS region. To test this hypothesis, we analyzed the impact of three mutations previously reported to disrupt structural domains of Prp18 (Supplementary Figure S7a): (i) a modified deletion of the conserved loop that disrupts growth above 34°C but binds Slu7p efficiently (*prp18-ΔCRt*, Δ(S187-I211)); (ii) a double point mutation in helix 2 that inhibits Slu7p binding (*prp18-h2*, R151E R152E) and (iii) a quadruple point mutation of four conserved residues in helix 5 that severely impacts growth at 30°C but retains the interaction with Slu7p (*prp18-h5*, D223K E224A K234A R235E) (40). All these mutants were expressed as GFP fusions and western blot analysis showed expression levels similar to the levels of the plasmid-borne wild-type Prp18p-GFP when cells were grown at 30°C (Supplementary Figure S7b). We then analyzed the impact of these mutations on the splicing of two pre-mRNAs that exhibited activation of non-consensus 3′SS in the absence of Prp18p: *NYV1* (CAU/ 3′SS) and *MUD1* (AUG/ 3′SS). Consistent with previous observations (40), the *prp18-h5* mutant displayed a severe growth defect at 30°C while the growth of the other two mutants was unaffected at this temperature, and all three mutants showed growth inhibition at 34 and 37°C (Figure 4B). This result indicates that the integrity of helix 5 is essential to achieve full functionality of Prp18p. Importantly, usage of the non-consensus 3′SS of *NYV1* and *MUD1* was significantly increased in all these mutants at 30°C (Figure 4C, D). Strikingly, usage of the *MUD1* AUG alternative 3′SS was even more pronounced in strains expressing the h5 and the *ΔCRt* mutants than in the *prp18*-null background (Figure 4d), suggesting that these mutants may have dominant phenotypes on the mechanism of 3′SS selection. We conclude that mutations within the α-helical domain of Prp18p can compromise the fidelity of selection of YAG 3′SS sequences. Importantly, mutations of the conserved loop of Prp18p promote usage of alternative 3′SS with sequences that differ from the consensus at positions –2 and –1. This result suggests that the role of the loop within the conserved region of Prp18p is not limited to binding nucleosides –3 and –4 of the 3′SS but extend to the entire 3′SS sequence. It is possible that these mutations may also impact the positioning of Prp8p α-finger domain, which directly interacts with the pyrimidine at position –3 of the 3′SS through Gln1594 (5). However, the α-finger domain of Prp8p is located away from Prp18p (Figure 4A), so it is unlikely that this is the major mechanism by which these mutations promote a loss of 3′SS fidelity mechanisms.

### Slu7p is necessary to recruit Prp18p but is not sufficient to fulfill its 3′SS fidelity functions

**Figure 5.**
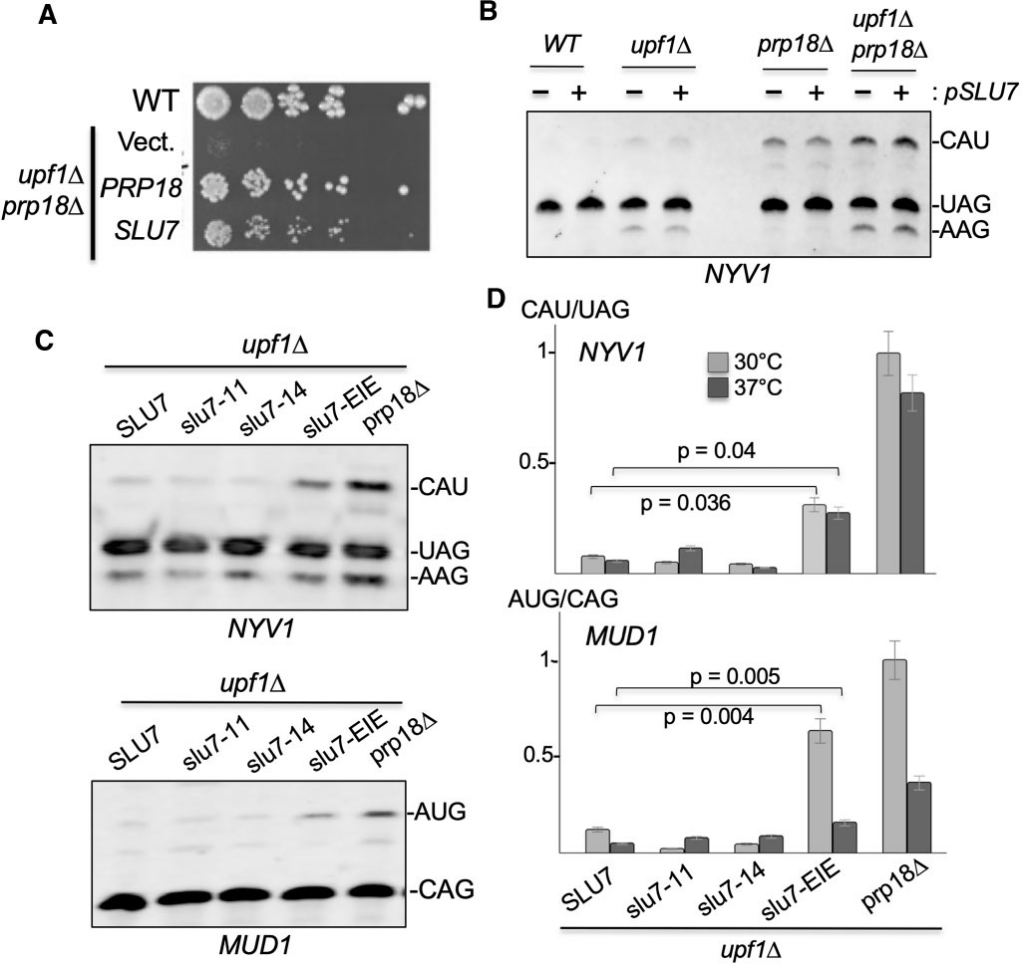
Slu7p is necessary to recruit Prp18p but not sufficient to fulfill its 3′SS fidelity functions. (**A**) Spot dilution growth assay for wild-type and *prp18*Δ*upf1*Δ strain complemented with Yep24 plasmids expressing *PRP18* or overexpressing *SLU7* or empty Yep24 (vector). (**B**) RT-PCR analysis of *NYV1* alternative 3′SS splicing in WT,*upf1*Δ, *prp18*Δ and *prp18*Δ*upf1*Δ strains transformed with a vector (–) or a plasmid overexpressing *SLU7* (+). (**C**) RT-PCR analysis of *NYV1* and *MUD1* alternative splicing in various *slu7* mutants. All strains are in a *upf1*Δ background. (**D**) Quantification of the ratio of usage of the major alternative 3′SS of *NYV1* and *MUD1* relative to the main 3′SS in the various *slu7* mutants. Legends as in Figure 4.

Some of the Prp18p mutations analyzed above impacted the interaction of Prp18p with Slu7p (e.g. h2), so we next asked if Slu7p may influence the ability of Prp18p to promote 3′SS fidelity, as Slu7p has been previously shown to influence 3′SS choice (41). Prior work has shown that an excess of Slu7p can restore the splicing defect of yeast splicing extracts depleted for Prp18p (15), suggesting that the role of Prp18p during splicing can be fulfilled solely by Slu7p. To test whether Slu7p can compensate for the loss of 3′SS fidelity detected in cells lacking Prp18p, we overexpressed Slu7p in the *prp18Δ* mutant and assayed growth and splicing phenotypes. Overexpression of Slu7p conferred a partial rescue of the growth defect observed in the *prp18Δupf1Δ* strain (Figure 5A), consistent with previous observations (40). However, overexpression of Slu7p in the *prp18Δupf1Δ* strain had no impact on usage of the annotated or alternative CAU or AAG sites the *NYV1* mRNA (Figure 5B). This result suggests that overexpressing Slu7p might rescue growth defects through enhanced splicing of a subset of intron-containing transcripts rather than by improving 3′SS fidelity. We next asked whether the recruitment of Prp18p by Slu7p is required to fulfill Prp18p's function in the fidelity of 3′SS selection. We analyzed *NYV1* and *MUD1* splicing in the previously described *slu7-11* (H75R, R243G, D267G), *slu7-14* (R173G, W210R, L297Q) and *slu7-EIE* (E215A-I216A-E217A) temperature-sensitive mutant strains (42). We found that the *slu7-EIE* allele is the only *slu7* mutant which exhibited significant usage of the non-canonical alternative 3′SS of *NYV1* or *MUD1* at 30°C (Figure 5C, D) or 37°C (Figure 5D). This result is consistent with previous data showing that the *slu7-EIE* mutation abolishes the interaction of Slu7p with Prp18p (43) and therefore prohibits the recruitment of Prp18p to the spliceosome, phenocopying the absence of Prp18p. We conclude that the recruitment of Prp18p by Slu7p is critical to promote the role of Prp18p in the fidelity of 3′SS selection, but that this role cannot be fulfilled by Slu7p alone, in contrast to previous *in vitro* results showing that the absence of Prp18p can be compensated by an excess of Slu7p (15). This result identifies a unique role for Prp18p in the fidelity of recognition of 3′SS sequences.

### Mutations at the Prp8p•Prp18p interface disable Prp18p 3′SS fidelity functions

Recent structural studies of yeast spliceosomes in various late stages of the spliceosome cycle have shown that Prp18p and Prp8p interact through the RNase H-like domain of Prp8p (Figure 4A) (5,44). Two *PRP8* mutant alleles, *prp8-121* and *prp8-123* were previously shown to confer increased splicing of reporter transcripts carrying point mutations within the 3′SS YAG motif(45). However, neither of these *prp8* mutants triggered alternative splicing at the non-canonical *NYV1* CAU 3′SS (Supplementary Figure S8). While it remains possible that these *prp8* mutations may affect splicing fidelity for other endogenous transcripts, this result demonstrates that the role of Prp18p in promoting 3′SS fidelity is likely to be distinct from the fidelity mechanism that is inhibited in the *prp8-121* and *prp8-12* alleles.

**Figure 6.**
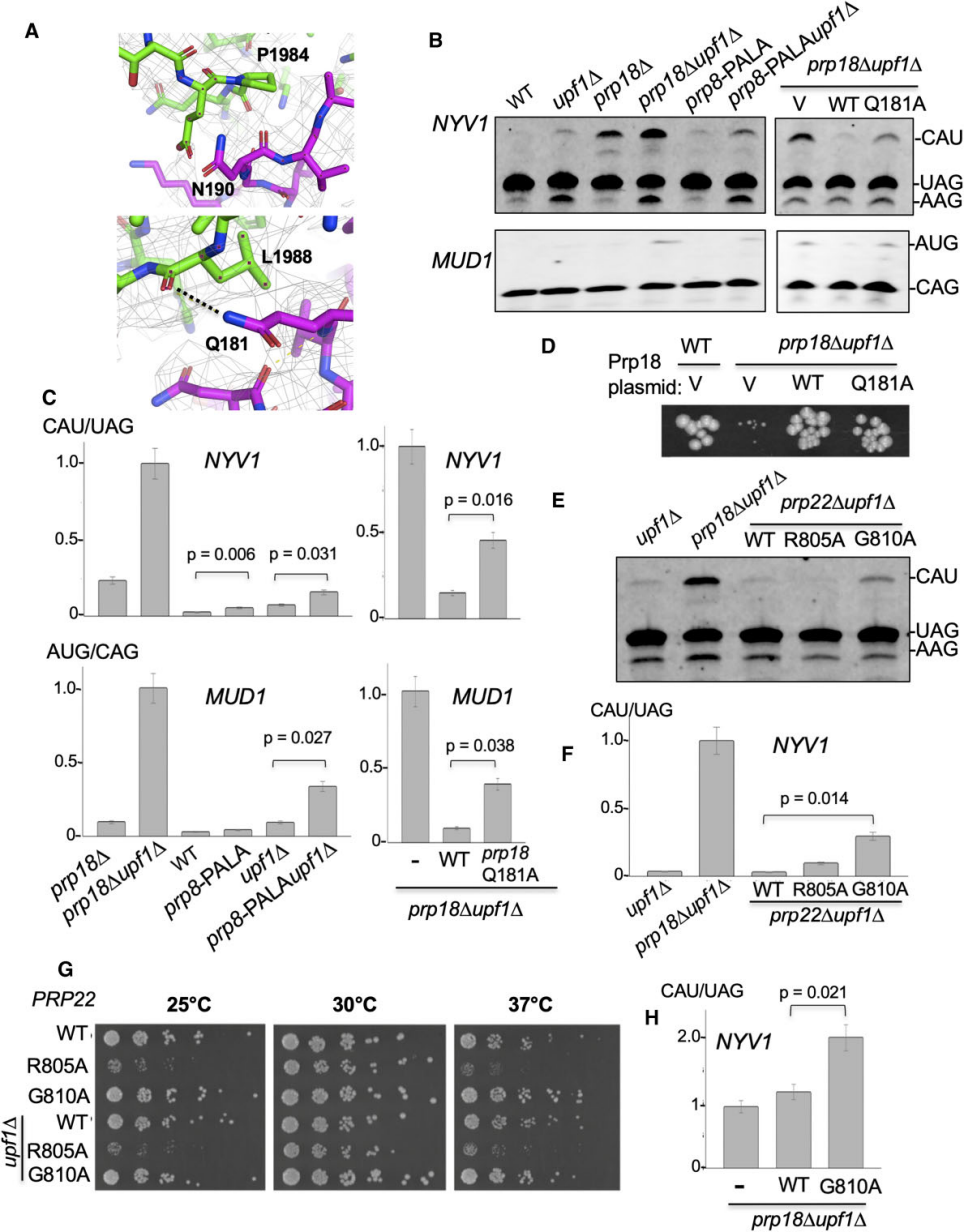
Prp8p and Prp22p synergize with Prp18p to promote 3′SS fidelity. (**A**) Structural details of catalytically activated step II spliceosome (60), left showing spatial proximity and potential interactions between Prp8p P1984 and Prp18p N190 (top), and Prp8p L1988 and Prp18p Q181 (bottom). Prp8 is colored with a green backbone while Prp18p is colored with a purple backbone. (**B**) RT-PCR analysis of *NYV1* and *MUD1* alternative splicing in a *prp8* mutant with a double point mutation P1984A and L1988A (*PALA*) in WT or *upf1*Δ background. WT, *upf1*Δ, *prp18*Δ and *prp18*Δ *upf1*Δ strains were included for reference. (**C**) Quantification of the ratio of usage of the major alternative 3′SS of *NYV1* and *MUD1* relative to the main 3′SS in the *prp8*-*PALA* mutant and other mutants analyzed in (B). Legends as in Figure 4. (**D**) Growth analysis of WT and *upf1*Δ*prp18*Δ transformed with a pUG35 vector (V) or a pUG35 plasmid containing WT *PRP18* (WT) or a Q181A mutation (*QA*). Strains were grown for 3 days at 30°C. RT-PCR analysis of *NYV1* splicing in wild-type, *prp18*Δ or *prp8* point mutant strains. (**E**) RT-PCR analysis of *NYV1* alternative splicing in wild-type, *prp18*Δ, or *prp18* point mutant strains. All strains were in a *upf1*Δ background. (**F**) Quantification of the ratio of usage of the major alternative 3′SS of *NYV1* for the strains analyzed in (E). Legends as in Figure 4. (**G**) Spot dilution growth assay showing *prp22*Δ null mutant yeast in either a wild-type (top three rows) or *upf1*Δ NMD mutant background (bottom three rows) carrying pRS315 plasmids expressing either wild- type *PRP22*, *prp22-R805A* or *prp22-G810A* alleles. Strains were grown to exponential phase in CSM-LEU media then serially diluted in 3-fold spot dilutions onto CSM-LEU plates and incubated at the indicated temperatures for 3 days. (**H**) Quantification of the ratio of usage of the major alternative 3′SS of *NYV1* for a *prp18*Δ*upf1*Δ strain transformed with an empty vector (V) or a pRS315 plasmid expressing *PRP22* (WT) or *prp22-G810A*. Legends as in Figure 4.

We next hypothesized that positioning of Prp18p by the Prp8p RNase H domain in the spliceosome active site would be critical to achieve fidelity. We identified two potential sites of interaction between Prp8p and Prp18p (Figure 6A). Prp8p P1984 is located near Prp18p N190 (Figure 6A, top) while the backbone amide of Prp8p L1988 forms a potential hydrogen bond with the side chain of Q181 of Prp18p (Figure 6A, bottom). To disrupt these predicted interactions, we introduced two mutations into the endogenous *PRP8* gene at P1984 and L1988 and assessed the impact of these mutations on 3′SS fidelity. The *prp8-P1984A-L1988A* (PALA) exhibited significantly increased usage of the alternative 3′-SS of *NYV1* and of *MUD1* (Figure 6B, C). To further evaluate the importance of the Prp8•Prp18p interface in 3′SS fidelity, we mutated the other side of the interface with an alanine substitution at residues Q181 of Prp18p (*prp18*-*QA* mutant). We found that the *prp18-QA* allele also resulted in a strong activation of the *NYV1* and *MUD1* alternative 3′SS (Figure 6B, C). Interestingly, this allele fully rescued the severe growth defect of the *prp18Δupf1Δ* double mutant (Figure 6D). In addition, the *prp18-QA* or *prp8-PALA* mutants did not exhibit any significant growth defects in liquid culture, whether or not NMD was active (Supplementary Figure S9). These growth assays suggest that the levels of unproductive alternatively spliced transcripts that accumulate in these fidelity mutants may not be sufficient to affect growth in standard laboratory conditions. We do not know, however if the 3′SS fidelity defects detected in these point mutants are as severe or global as those observed in a strain that lacks Prp18p completely, as we did not perform RNA-seq analysis of these mutants. So it is possible that the 3′SS fidelity defects detected in these mutants affected only a few specific transcripts such as *MUD1* and *NYV1*, which may explain the lack of growth phenotypes. However, we conclude that the Prp18•Prp8 interface is critical for the fidelity of 3′SS recognition by the spliceosome and to prevent usage of some non-consensus 3′SS by the spliceosome.

### Prp22p, but not Prp43p, acts downstream of Prp18p to proofread erroneous selection of non-consensus 3′ splice sites

We next explored whether other splicing factors may assist or synergize Prp18p function in promoting 3′SS fidelity. We began with Prp17p, which stabilizes the C* complex following remodeling by Prp16p but preceding exon ligation (44), and Cwc21p, which has also been shown to influence 3′SS selection (46). RT-PCR analysis of *prp17Δ* and *cwc21Δ* null mutants revealed no increased usage of the non-AG 3′SS of *NYV1* (Supplementary Figure S10). We next analyzed mutants of the RNA helicase Prp22p due to its known role in proofreading splicing (13) and mediating alternative 3′ SS selection (12). In addition, structural studies have suggested that the ability of Prp22p to proofread *bona fide* 3′SS sequences might be linked to its ability to sense the stability of the docked 3′SS engaged in non-Watson-Crick interactions with the 5′SS (5). Previous work found increased usage of a non-consensus RAG 3′SS in splicing reporters in two *prp22* mutants harboring mutations within the conserved motif VI, *prp22-R805A* and *prp22-G810A* (13,47), so we tested the impact of these two mutants on splicing fidelity. Strikingly, we found that the *prp22-G810A* allele increased usage of the *NYV1* CAU alternative 3′SS when this mutant was grown at the permissive temperature of 30°C (Figure 6E, F). By contrast, the *prp22-R805A* mutant did not appear to impact *NYV1* splicing fidelity (Figure 6E, F) despite having much stronger growth defects at reduced and elevated temperatures relative to *prp22-*G810A (Figure 6E–G). The growth of these two mutants at different temperatures (Figure 6G) was consistent with previous reports (47), and inactivation of *UPF1* had no further effect on cellular growth of these mutants (Figure 6G).

To test whether the role of Prp22p in 3′SS fidelity is also shared with other late splicing factors, we analyzed mutants of the DEAH-box RNA helicase Prp43p, which facilitates the disassembly of the U2, U5, and U6 snRNPs from the excised intron lariat (48,49). The *prp43-Q423N* and *prp43-Q423E* motif VI mutations result in cold sensitivity *in vivo* and the *prp43-Q423E* mutation was shown to increase usage of a cryptic 3′SS in a splicing reporter (50), suggesting a role for Prp43p in the fidelity of 3′SS selection. We found no evidence for increased usage of the *NYV1* alternative 3′SS in either of these mutants in the *upf1*Δ background at either the permissive or non-permissive temperatures (Supplementary Figure S11), suggesting that Prp43p does not contribute to the mechanisms of 3′SS fidelity governed by Prp18p and Prp22p.

The finding that alternatively spliced *NYV1* transcripts using the non-canonical 3′SS can be detected in both Prp18p and Prp22p (but not Prp43p) mutants suggests that the two proteins may act sequentially in promoting the fidelity of 3′SS selection, with Prp18p playing a primary role in promoting the stable docking of consensus 3′SS sequences and Prp22p playing a downstream proofreading role, as suggested previously (13). To test this hypothesis, we expressed the *prp22-G810A* mutant in the *upf1Δprp18Δ* background, with the idea that expressing this mutant should be epistatic to the absence of Prp18p if these two proteins act at the same step. Strikingly, expressing the *prp22-G810A* mutant in the *upf1Δprp18Δ* background resulted in an additive effect on the accumulation of the *NYV1* CAU alt.3′SS (Figure 6H). This result demonstrates that Prp18p and Prp22p function independently and sequentially to promote the fidelity of 3′SS selection. Our results suggest that Prp18p promotes docking of consensus 3′SS sequences at a minimum distance of 10 bp from the branchpoint, while Prp22p assumes a downstream proofreading role to reject non YAG 3′SS docked in the active site but with suboptimal interactions with the 5′SS and/or branchpoint sequences. The rejection of these substrates by Prp22p would be consistent with the classical kinetic proofreading mechanism proposed for splicing helicases (51).

## Discussion

In this study, we show that Prp18p plays a global role in promoting the selection of consensus 3′SS sequences by the spliceosome *in vivo* in *S. cerevisiae*. While structural studies had shown that Prp18p promotes docking of the 3′SS in the spliceosome active site, our study provides for the first time evidence for genome-wide loss of splicing fidelity *in vivo* in the absence of Prp18p. In particular we demonstrate a global relaxed fidelity of the spliceosome towards non-YAG 3′ SS sequences. To our knowledge, this is the first report of such widespread usage of non-consensus 3′ SS sequences *in vivo*. The loss of fidelity we detect is mainly specific to the loss of Prp18p, as it is not detected in most other splicing mutants directly implicated in the second splicing step, including *prp17* and some *prp8* mutants previously described to affect 3′SS fidelity (45). However, relaxed fidelity is also triggered by mutations that prevent recruitment of Prp18p (such as *slu7-EIE*), or perturb its interactions in the spliceosome active site (such as mutations at the Prp8p-Prp18p interface; Figure 6). It is possible that this 3′SS sequence fidelity mechanism also involves the newly identified second step factor Fyv6p, as inactivation of Fyv6p was found to result in an increased usage of upstream 3′SS of a reporter transcript and of the endogenous *SUS1* pre-mRNA (11). Identification of a potential role of Fyv6p in 3′SS sequence fidelity awaits further investigations. Lastly, we demonstrate that the fidelity function of Prp18p is enhanced by the proofreading activity of Prp22p, as shown by the additive effects of inactivating Prp18p and expressing dominant negative versions of Prp22p. While this fidelity function was previously demonstrated using reporter RNAs (13), we show that it operates i*n vivo* on endogenous RNA substrates, and that it functions sequentially after Prp18p. Therefore, 3′SS sequence fidelity is a multi-step process in which Prp18p acts to promote fidelity of selection of canonical 3′SS sequences, while Prp22p proofreads non-consensus 3′SS sequences docked in the active site that may have escaped the Prp18p primary selection mechanism. The ability of Prp22p to proofread these splicing events might be linked to a more unstable binding of these suboptimal 3′SS with the 5′SS or branchpoint in the spliceosome active site, consistent with the kinetic proofreading model proposed for splicing helicases (51).

Alternative, non-consensus 3′SS used in the absence of Prp18p are highly diverse in their sequence, which raises the question why specific sites are being used as opposed to others which are very similar and located nearby. We found that several intronic and pseudo-exonic features such as the distance from the branchpoint to the 3′SS, the presence of poly(U) tracts upstream the alternative and annotated sites, adenosine content in the exons or pseudo-exons, and accessibility of the site within RNA secondary structure likely combine to dictate the efficiency of alternative 3′SS usage. After the first chemical step of splicing, the spliceosome needs to dock the 3′SS in the active site. This docking process might be slower in the absence of Prp18p, which may provide opportunity for non-canonical 3′SS sequences located upstream from the normal 3′SS to dock prematurely in the spliceosome active site and compete with the *bona fide* 3′SS (Figure 3A, B). We propose a model wherein in the absence of Prp18p, non-canonical 3′SS sequences are more likely to engage in promiscuous interactions with the first intronic nucleotide à la Parker and Siliciano (3) and position the 3′SS for ligation as shown in spliceosome structures (5) (Supplementary Figure S12a). In agreement with this model, a previous study showed that introducing point mutations in the G1 and G-1 of the 3′SS can trigger activation of a cryptic upstream 3′SS that recapitulate these non-Watson-Crick interactions (4). In light of the frequent activation of HAU alternative 3′SS sequences, we propose that in the absence of Prp18p, there may be increased flexibility in the active site which allows for an alternative base-pairing with the 3′SS U**_-1_** and the 5′SS G**_+1_** (Supplementary Figure S12b). Substituting a cytosine or adenosine at the last position of the 3′SS would introduce a steric clash or incompatible hydrogen bond donor/acceptor arrangements that would disrupt the non-Watson Crick interaction with G1 (Supplementary Figure S12c, d), providing a rationale for the preference for Us we observe at the last position of non-G alternative 3′ SS.

Lastly, it is important to consider the potential conservation of the role of Prp18p in splicing fidelity. Prp18p (or PRPF18 in higher eukaryotes) was found to be present in the last eukaryotic common ancestor (LECA), pointing to an ancient role in splicing (52). However some organisms may have evolved in their requirement for Prp18p. For instance, different budding yeast species have been shown to exhibit different ranges of BP-3′SS distances, with the salt-tolerant species *Debaryomyces hansenii* exhibiting the shortest predicted BP-3′SS distances of predominantly 7–8 nt (27). Because this type of substrate was shown not to require Prp18p for efficient splicing, it is possible that the effect of inactivating Prp18p in this organism might be less severe than in *S. cerevisiae*. In addition, Prp18p (and Slu7p) are not universally present in eukaryotes, as shown by the reduced spliceosome of the red algae *Cyanidioschyzon merolae*, which lacks these two proteins entirely (53). This observation begs the question of which proteins might be primarily involved in the mechanism of 3′SS fidelity, and whether the role of Prp18p in this process has been replaced by stronger interactions between Prp8p and the 3′SS, or by a more prominent role for Prp22p. In mammalian transcriptomes, 3′SS recognition is somewhat more complex than in *S. cerevisiae* considering the length of intronic sequences, which are much longer than in *S. cerevisiae*. hSlu7, the human homolog of yeast Slu7p, has been shown to be required for correct 3′SS choice in mammalian cells (54). Prp18p is also conserved in mammalian cells and was shown to be required for the 2^nd^ step of splicing *in vitro* (55). However, biochemical and structural characterization of human C* and P spliceosomes showed the absence of the human Prp18p homologue PRPF18 (56–58). In the absence of PRPF18, additional, human specific factors are present, which facilitate the second splicing step (56). On one hand these observations may argue against a conserved role for Prp18p in promoting splicing fidelity in mammalian splicing. On the other hand it is possible that PRPF18 is necessary to promote splicing efficiency and fidelity for specific transcripts. This hypothesis is supported by the observation that the role for PRPF18 in splicing *in vitro* was established using a β-globin pre-mRNA (55), which differs from the MINX pre-mRNA used in the later biochemical and structural studies of human spliceosomes (56,57,59). Therefore it is possible that the role of Prp18p in splicing fidelity might be conserved in humans but restricted to specific transcripts or cell types. This would be reminiscent of the situation in yeast, where we have shown that the loss of Prp18p impacts much more severely non-ribosomal protein gene introns. Therefore, a full answer to the question of the role of PRPF18 in splicing fidelity awaits further genomic analyses in human systems.

## Supplementary Material

gkad968_Supplemental_Files

## Data Availability

All raw and processed data from this study have been submitted to the NCBI Gene Expression Omnibus (GEO; http://www.ncbi.nlm.nih.gov/geo/) under accession number GSE131797. All scripts for the COMPASS read processing pipeline used in this publication, are available on Zenodo: https://doi.org/10.5281/zenodo.8387420.

## References

[1] Stanley R.F. , Abdel-Wahab O. Dysregulation and therapeutic targeting of RNA splicing in cancer. Nat Cancer. 2022; 3:536–546. 35624337 10.1038/s43018-022-00384-z PMC9551392

[2] Wu S. , Romfo C.M., Nilsen T.W., Green M.R. Functional recognition of the 3’ splice site AG by the splicing factor U2AF35. Nature. 1999; 402:832–835. 10617206 10.1038/45590

[3] Parker R. , Siliciano P.G. Evidence for an essential non-Watson–Crick interaction between the first and last nucleotides of a nuclear pre-mRNA intron. Nature. 1993; 361:660–662. 8437627 10.1038/361660a0

[4] Chanfreau G. , Legrain P., Dujon B., Jacquier A. Interaction between the first and last nucleotides of pre-mRNA introns is a determinant of 3’ splice site selection in S. cerevisiae. Nucleic Acids Res. 1994; 22:1981–1987. 8029003 10.1093/nar/22.11.1981 PMC308110

[5] Wilkinson M.E. , Fica S.M., Galej W.P., Norman C.M., Newman A.J., Nagai K. Postcatalytic spliceosome structure reveals mechanism of 3′–splice site selection. Science. 2017; 358:1283–1288. 29146871 10.1126/science.aar3729 PMC5808836

[6] Liu S. , Li X., Zhang L., Jiang J., Hill R.C., Cui Y., Hansen K.C., Zhou Z.H., Zhao R. Structure of the yeast spliceosomal postcatalytic P complex. Science. 2017; 358:1278–1283. 29146870 10.1126/science.aar3462 PMC5828012

[7] Schwer B. , Guthrie C. A conformational rearrangement in the spliceosome is dependent on PRP16 and ATP hydrolysis. EMBO J. 1992; 11:5033–5039. 1464325 10.1002/j.1460-2075.1992.tb05610.x PMC556981

[8] Wilkinson M.E. , Fica S.M., Galej W.P., Nagai K. Structural basis for conformational equilibrium of the catalytic spliceosome. Mol. Cell. 2021; 81:1439–1452. 33705709 10.1016/j.molcel.2021.02.021 PMC8022279

[9] Ohrt T. , Odenwälder P., Dannenberg J., Prior M., Warkocki Z., Schmitzová J., Karaduman R., Gregor I., Enderlein J., Fabrizio P. et al. Molecular dissection of step 2 catalysis of yeast pre-mRNA splicing investigated in a purified system. RNA. 2013; 19:902–915. 23685439 10.1261/rna.039024.113 PMC3683925

[10] Umen J.G. , Guthrie C. Prp16p, Slu7p, and Prp8p interact with the 3’ splice site in two distinct stages during the second catalytic step of pre-mRNA splicing. RNA. 1995; 1:584–597. 7489518 PMC1369303

[11] Lipinski K.A. , Senn K.A., Zeps N.J., Hoskins A.A. Biochemical and genetic evidence supports Fyv6 as a second-step splicing factor in *Saccharomyces cerevisiae*. RNA. 2023; 29:1792–1802. 37625852 10.1261/rna.079607.123 PMC10578475

[12] Semlow D.R. , Blanco M.R., Walter N.G., Staley J.P. Spliceosomal DEAH-Box ATPases remodel pre-mRNA to activate alternative splice sites. Cell. 2016; 164:985–998. 26919433 10.1016/j.cell.2016.01.025 PMC4979991

[13] Mayas R.M. , Maita H., Staley J.P. Exon ligation is proofread by the DExD/H-box ATPase Prp22p. Nat. Struct. Mol. Biol. 2006; 13:482–490. 16680161 10.1038/nsmb1093 PMC3729281

[14] Kawashima T. , Pellegrini M., Chanfreau G.F. Nonsense-mediated mRNA decay mutes the splicing defects of spliceosome component mutations. RNA. 2009; 15:2236–2247. 19850912 10.1261/rna.1736809 PMC2779665

[15] Zhang X. , Schwer B. Functional and physical interaction between the yeast splicing factors Slu7 and Prp18. Nucleic Acids Res. 1997; 25:2146–2152. 9153314 10.1093/nar/25.11.2146 PMC146706

[16] Brys A. , Schwer B. Requirement for SLU7 in yeast pre-mRNA splicing is dictated by the distance between the branchpoint and the 3’ splice site. RNA. 1996; 2:707–717. 8756413 PMC1369409

[17] Crotti L.B. , Bacikova D., Horowitz D.S. The Prp18 protein stabilizes the interaction of both exons with the U5 snRNA during the second step of pre-mRNA splicing. Genes Dev. 2007; 21:1204–1216. 17504938 10.1101/gad.1538207 PMC1865492

[18] Crotti L.B. , Horowitz D.S. Exon sequences at the splice junctions affect splicing fidelity and alternative splicing. Proc. Natl. Acad. Sci. U.S.A. 2009; 106:18954–18959. 19855008 10.1073/pnas.0907948106 PMC2776460

[19] Kawashima T. , Douglass S., Gabunilas J., Pellegrini M., Chanfreau G.F. Widespread use of non-productive alternative splice sites in *Saccharomyces cerevisiae*. PLoS Genet. 2014; 10:e1004249. 24722551 10.1371/journal.pgen.1004249 PMC3983031

[20] Gabunilas J. , Chanfreau G. Splicing-mediated autoregulation modulates Rpl22p expression in *Saccharomyces cerevisiae*. PLoS Genet. 2016; 12:e1005999. 27097027 10.1371/journal.pgen.1005999 PMC4838235

[21] Roy K. , Chanfreau G. Stress-induced nuclear RNA degradation pathways regulate yeast bromodomain factor 2 to promote cell survival. PLoS Genet. 2014; 10:e1004661. 25232960 10.1371/journal.pgen.1004661 PMC4169253

[22] Roy K. , Gabunilas J., Gillespie A., Ngo D., Chanfreau G.F. Common genomic elements promote transcriptional and DNA replication roadblocks. Genome Res. 2016; 26:1363–1375. 27540088 10.1101/gr.204776.116 PMC5052057

[23] Gibson D.G. , Young L., Chuang R.-Y., Venter J.C., Hutchison C.A., Smith H.O. Enzymatic assembly of DNA molecules up to several hundred kilobases. Nat. Methods. 2009; 6:343–345. 19363495 10.1038/nmeth.1318

[24] Gillespie A. , Gabunilas J., Jen J.C., Chanfreau G.F. Mutations of EXOSC3/Rrp40p associated with neurological diseases impact ribosomal RNA processing functions of the exosome in *S. cerevisiae*. RNA. 2017; 23:466–472. 28053271 10.1261/rna.060004.116 PMC5340910

[25] Defenouillère Q. , Zhang E., Namane A., Mouaikel J., Jacquier A., Fromont-Racine M. Rqc1 and Ltn1 prevent C-terminal alanine-threonine tail (CAT-tail)-induced protein aggregation by efficient recruitment of Cdc48 on stalled 60S subunits. J. Biol. Chem. 2016; 291:12245–12253. 27129255 10.1074/jbc.M116.722264 PMC4933273

[26] Dobin A. , Davis C.A., Schlesinger F., Drenkow J., Zaleski C., Jha S., Batut P., Chaisson M., Gingeras T.R. STAR: ultrafast universal RNA-seq aligner. Bioinformatics. 2013; 29:15–21. 23104886 10.1093/bioinformatics/bts635 PMC3530905

[27] Gahura O. , Hammann C., Valentová A., Půta F., Folk P. Secondary structure is required for 3′ splice site recognition in yeast. Nucleic Acids Res. 2011; 39:9759–9767. 21893588 10.1093/nar/gkr662 PMC3239191

[28] Meyer M. , Plass M., Perez-Valle J., Eyras E., Vilardell J. Deciphering 3’ss selection in the yeast genome reveals an RNA thermosensor that mediates alternative splicing. Mol. Cell. 2011; 43:1033–1039. 21925391 10.1016/j.molcel.2011.07.030

[29] Lorenz R. , Bernhart S.H., Höner zu Siederdissen C., Tafer H., Flamm C., Stadler P.F., Hofacker I.L. ViennaRNA Package 2.0. Algorith. Mol. Biol. 2011; 6:26. 10.1186/1748-7188-6-26 PMC3319429 22115189

[30] Ma P. , Xia X. Factors affecting splicing strength of yeast genes. Comp. Funct. Genomics. 2011; 2011:212146. 22162666 10.1155/2011/212146 PMC3226532

[31] Weathers I. , Gabunilas J., Samson J., Roy K., Chanfreau G.F. Protocol for high-resolution mapping of splicing products and isoforms by RT-PCR using fluorescently labeled primers. STAR Protoc. 2020; 1:100140. 33377034 10.1016/j.xpro.2020.100140 PMC7757285

[32] Ares Jr. M. , Grate L., Pauling M.H. A handful of intron-containing genes produces the lion's share of yeast mRNA. RNA. 1999; 5:1138–1139. 10496214 10.1017/s1355838299991379 PMC1369836

[33] Pleiss J.A. , Whitworth G.B., Bergkessel M., Guthrie C. Rapid, transcript-specific changes in splicing in response to environmental stress. Mol. Cell. 2007; 27:928–937. 17889666 10.1016/j.molcel.2007.07.018 PMC2081968

[34] Aslanzadeh V. , Huang Y., Sanguinetti G., Beggs J.D. Transcription rate strongly affects splicing fidelity and cotranscriptionality in budding yeast. Genome Res. 2018; 28:203–213. 29254943 10.1101/gr.225615.117 PMC5793784

[35] Gould G.M. , Paggi J.M., Guo Y., Phizicky D.V., Zinshteyn B., Wang E.T., Gilbert W.V., Gifford D.K., Burge C.B. Identification of new branch points and unconventional introns in Saccharomyces cerevisiae. RNA. 2016; 22:1522–1534. 27473169 10.1261/rna.057216.116 PMC5029451

[36] Schreiber K. , Csaba G., Haslbeck M., Zimmer R. Alternative splicing in next generation sequencing data of *Saccharomyces cerevisiae*. PLoS One. 2015; 10:e0140487. 26469855 10.1371/journal.pone.0140487 PMC4607428

[37] Plass M. , Codony-Servat C., Ferreira P.G., Vilardell J., Eyras E. RNA secondary structure mediates alternative 3’ss selection in Saccharomyces cerevisiae. RNA. 2012; 18:1103–1115. 22539526 10.1261/rna.030767.111 PMC3358634

[38] Bacikova D. , Horowitz D.S. Genetic and functional interaction of evolutionarily conserved regions of the Prp18 protein and the U5 snRNA. Mol. Cell. Biol. 2005; 25:2107–2116. 15743809 10.1128/MCB.25.6.2107-2116.2005 PMC1061626

[39] Jiang J. , Horowitz D.S., Xu R.-M. Crystal structure of the functional domain of the splicing factor Prp18. Proc. Natl. Acad. Sci. U.S.A. 2000; 97:3022–3027. 10737784 10.1073/pnas.97.7.3022 PMC16185

[40] Bacikova D. , Horowitz D.S. Mutational analysis identifies two separable roles of the *Saccharomyces cerevisiae* splicing factor Prp18. RNA. 2002; 8:1280–1293. 12403466 10.1017/s1355838202023099 PMC1370337

[41] Frank D. , Guthrie C. An essential splicing factor, SLU7, mediates 3’ splice site choice in yeast. Genes Dev. 1992; 6:2112–2124. 1427075 10.1101/gad.6.11.2112

[42] Aronova A. , Bacíková D., Crotti L.B., Horowitz D.S., Schwer B. Functional interactions between Prp8, Prp18, Slu7, and U5 snRNA during the second step of pre-mRNA splicing. RNA. 2007; 13:1437–1444. 17626844 10.1261/rna.572807 PMC1950762

[43] James S.A. , Turner W., Schwer B. How Slu7 and Prp18 cooperate in the second step of yeast pre-mRNA splicing. RNA. 2002; 8:1068–1077. 12212850 10.1017/s1355838202022033 PMC1370317

[44] Fica S.M. , Oubridge C., Galej W.P., Wilkinson M.E., Bai X.-C., Newman A.J., Nagai K. Structure of a spliceosome remodelled for exon ligation. Nature. 2017; 542:377–380. 28076345 10.1038/nature21078 PMC5321579

[45] Umen J.G. , Guthrie C. Mutagenesis of the yeast gene PRP8 reveals domains governing the specificity and fidelity of 3’ splice site selection. Genetics. 1996; 143:723–739. 8725222 10.1093/genetics/143.2.723 PMC1207332

[46] Gautam A. , Grainger R.J., Vilardell J., Barrass J.D., Beggs J.D. Cwc21p promotes the second step conformation of the spliceosome and modulates 3′ splice site selection. Nucleic Acids Res. 2015; 43:3309. 25740649 10.1093/nar/gkv159 PMC4381068

[47] Schwer B. , Meszaros T. RNA helicase dynamics in pre-mRNA splicing. EMBO J. 2000; 19:6582–6591. 11101530 10.1093/emboj/19.23.6582 PMC305851

[48] Fourmann J.-B. , Tauchert M.J., Ficner R., Fabrizio P., Lührmann R. Regulation of Prp43-mediated disassembly of spliceosomes by its cofactors Ntr1 and Ntr2. Nucleic Acids Res. 2017; 45:4068–4080. 27923990 10.1093/nar/gkw1225 PMC5397206

[49] Tsai R.-T. , Fu R.-H., Yeh F.-L., Tseng C.-K., Lin Y.-C., Huang Y., Cheng S.-C. Spliceosome disassembly catalyzed by Prp43 and its associated components Ntr1 and Ntr2. Genes Dev. 2005; 19:2991–3003. 16357217 10.1101/gad.1377405 PMC1315403

[50] Mayas R.M. , Maita H., Semlow D.R., Staley J.P. Spliceosome discards intermediates via the DEAH box ATPase Prp43p. Proc. Natl. Acad. Sci. U.S.A. 2010; 107:10020–10025. 20463285 10.1073/pnas.0906022107 PMC2890470

[51] Staley J.P. , Guthrie C. Mechanical devices of the spliceosome: motors, clocks, springs, and things. Cell. 1998; 92:315–326. 9476892 10.1016/s0092-8674(00)80925-3

[52] Vosseberg J. , Stolker D., von der Dunk S.H.A., Snel B. Integrating phylogenetics with intron positions illuminates the origin of the complex spliceosome. Mol. Biol. Evol. 2023; 40:msad011. 36631250 10.1093/molbev/msad011 PMC9887622

[53] Black C.S. , Whelan T.A., Garside E.L., Macmillan A.M., Fast N.M., Rader S.D. Spliceosome assembly and regulation: insights from analysis of highly reduced spliceosomes. RNA. 2023; 29:531–550. 36737103 10.1261/rna.079273.122 PMC10158995

[54] Chua K. , Reed R. The RNA splicing factor hSlu7 is required for correct 3’ splice-site choice. Nature. 1999; 402:207–210. 10647016 10.1038/46086

[55] Horowitz D.S. , Krainer A.R. A human protein required for the second step of pre-mRNA splicing is functionally related to a yeast splicing factor. Genes Dev. 1997; 11:139–151. 9000057 10.1101/gad.11.1.139

[56] Fica S.M. , Oubridge C., Wilkinson M.E., Newman A.J., Nagai K. A human postcatalytic spliceosome structure reveals essential roles of metazoan factors for exon ligation. Science. 2019; 363:710–714. 30705154 10.1126/science.aaw5569 PMC6386133

[57] Ilagan J.O. , Chalkley R.J., Burlingame A.L., Jurica M.S. Rearrangements within human spliceosomes captured after exon ligation. RNA. 2013; 19:400–412. 23345524 10.1261/rna.034223.112 PMC3677250

[58] Dybkov O. , Preußner M., Ayoubi L.E., Feng V.Y., Harnisch C., Merz K., Leupold P., Yudichev P., Agafonov D.E., Will C.L. et al. Regulation of 3′ splice site selection after step 1 of splicing by spliceosomal C* proteins. Sci. Adv. 2023; 9:eadf178. 10.1126/sciadv.adf1785 PMC9984181 36867703

[59] Bertram K. , Agafonov D.E., Liu W.T., Dybkov O., Will C.L., Hartmuth K., Urlaub H., Kastner B., Stark H., Lührmann R. Cryo-EM structure of a human spliceosome activated for step 2 of splicing. Nature. 2017; 542:318–323. 28076346 10.1038/nature21079

[60] Yan C. , Wan R., Bai R., Huang G., Shi Y. Structure of a yeast step II catalytically activated spliceosome. Science. 2017; 355:149–155. 27980089 10.1126/science.aak9979

